# Population Attributable Fractions of Modifiable Risk Factors for Nonsyndromic Orofacial Clefts: A Prospective Cohort Study From the Japan Environment and Children’s Study

**DOI:** 10.2188/jea.JE20190347

**Published:** 2021-04-05

**Authors:** Yukihiro Sato, Eiji Yoshioka, Yasuaki Saijo, Toshinobu Miyamoto, Kazuo Sengoku, Hiroshi Azuma, Yusuke Tanahashi, Yoshiya Ito, Sumitaka Kobayashi, Machiko Minatoya, Yu Ait Bamai, Keiko Yamazaki, Sachiko Itoh, Chihiro Miyashita, Atsuko Araki, Reiko Kishi, the Japan Environment and Children’s Study (JECS) Group

**Affiliations:** 1 Division of Public Health and Epidemiology, Department of Social Medicine, Asahikawa Medical University, Hokkaido, Japan; 2 Department of Obstetrics and Gynecology, Asahikawa Medical University, Hokkaido, Japan; 3 Department of Pediatrics, Asahikawa Medical University, Hokkaido, Japan; 4 Faculty of Nursing, Japanese Red Cross Hokkaido College of Nursing, Hokkaido, Japan; 5 Center for Environmental and Health Sciences, Hokkaido University, Hokkaido, Japan; 6 Faculty of Health Sciences, Hokkaido University, Hokkaido, Japan

**Keywords:** orofacial clefts, cohort study, population attributable fraction, cleft lip with or without cleft palate

## Abstract

**Background:**

Population impact of modifiable risk factors on orofacial clefts is still unknown. This study aimed to estimate population attributable fractions (PAFs) of modifiable risk factors for nonsyndromic cleft lip with or without cleft palate (CL±P) and cleft palate only (CP) in Japan.

**Methods:**

We conducted a prospective cohort study using data from the Japan Environment and Children’s Study, which recruited pregnant women from 2011 to 2014. We estimated the PAFs of maternal alcohol consumption, psychological distress, maternal active and passive smoking, abnormal body mass index (BMI) (<18.5 and ≥25 kg/m^2^), and non-use of a folic acid supplement during pregnancy for nonsyndromic CL±P and CP in babies.

**Results:**

A total of 94,174 pairs of pregnant women and their single babies were included. Among them, there were 146 nonsyndromic CL±P cases and 41 nonsyndromic CP cases. The combined adjusted PAF for CL±P of the modifiable risk factors excluding maternal alcohol consumption was 34.3%. Only maternal alcohol consumption was not associated with CL±P risk. The adjusted PAFs for CL±P of psychological distress, maternal active and passive smoking, abnormal BMI, and non-use of a folic acid supplement were 1.4% (95% confidence interval [CI], −10.7 to 15.1%), 9.9% (95% CI, −7.0 to 26.9%), 10.8% (95% CI, −9.9 to 30.3%), 2.4% (95% CI, −7.5 to 14.0%), and 15.1% (95% CI, −17.8 to 41.0%), respectively. We could not obtain PAFs for CP due to the small sample size.

**Conclusions:**

We reported the population impact of the modifiable risk factors on CL±P, but not CP. This study might be useful in planning the primary prevention of CL±P.

## INTRODUCTION

Orofacial clefts are a common congenital anomaly, with approximately 1 case per 700 live births.^1^^,^^2^ Especially Chinese and Japanese has a high incidence rate of orofacial clefts compared with other ethnicities.^1^ Orofacial clefts are classified into two etiologically distinct groups, which result from inadequate formations during embryogenesis development: cleft lip with or without cleft palate (CL±P) and cleft palate only (CP). This anomaly can be part of a syndrome or malformations; therefore, there are nonsyndromic (without other congenital anomalies and any syndrome) and syndromic cases. Individuals with orofacial clefts have increased risk of disabilities^3^ and the higher economic burdens compared with nonaffected individuals.^4^ Preventive efforts on orofacial clefts are still desired.

Previous reviews revealed the potential lifestyle and environmental risk factors for nonsyndromic orofacial clefts.^1^^,^^5^ Reviews showed that maternal active smoking and passive smoking increases the risk for orofacial clefts by approximately 1.4 times and 2.1 times, respectively.^6^^–^^9^ Besides, maternal obesity is also associated with approximately 1.2 times risk.^10^^–^^12^ A review suggested the associations between stressful event and CL±P.^9^ Although maternal alcohol consumption during the pregnancy is recognized as a potential risk factor for orofacial clefts, there is no consistent evidence.^9^^,^^13^ Folic acid supplementation is a potential preventive factor for both CL±P and CP.^14^^,^^15^

In planning the primary prevention of orofacial clefts, it is useful to assess the public health impact of a risk factor on populations, using the population attributable fraction (PAF).^16^ To our best knowledge, only two studies reported the PAFs of orofacial clefts. The secondary data analysis study reported that the PAF of smoking for orofacial clefts was 6.1% in the United States in 2010.^17^ PAF denotes the fraction of cases that would not have occurred if exposure did not occur, and the association is causal.^18^ Thus, the PAF indicates that the annual potentially preventable proportion of orofacial clefts by the smoking ban was 6.1% in the United States (430 cases).^17^ A case-control study conducted in the United States also reported PAFs of lack of folic acid supplementation, maternal smoking, alcohol consumption, and obesity for both CL±P and CP.^19^ The PAFs of maternal smoking for CL±P and CP were 3.99% and 3.38%, respectively. Insufficient folic acid supplementation was associated with CL±P only, with a PAF of 3.34%. The PAF of maternal alcohol consumption was reported on CP only, which was 0.78%. Obesity was not associated with both CL±P and CP. However, these studies used secondary and case-control data. For example, the case-control study used the risk factors potentially reported after exposures occurring more than 2 years.^19^ To reduce the recall bias and verify the validity of the results, a pregnancy cohort study through which details on the relevant parameters are gathered in advance of the outcome is needed. This prospective nationwide birth cohort study aimed to examine the association of modifiable risk factors including psychological distress, maternal alcohol consumption, maternal active smoking, maternal passive smoking, abnormal body mass index (BMI), and non-use of a folic acid supplement, with nonsyndromic CL±P and CP and estimate the PAFs from the Japan Environment and Children’s Study (JECS).

## METHODS

### Ethical approval

The JECS protocol was approved by the Institutional Review Board on epidemiological studies of the Ministry of the Environment and ethics committees of all participating institutions. The JECS was conducted in accordance to the Declaration of Helsinki and other nationally valid regulations. All participating mothers and fathers had provided written informed consent.

### Data sources and participants

We used the dataset of jecs-an-20180131 from the JECS, the details of which are published elsewhere.^20^ The JECS is an ongoing nationwide birth cohort study in Japan that aims to identify environmental factors of children’s health and development. The 15 regional centers were selected to cover the Japanese geographical areas from the north, Hokkaido, to the south, Okinawa. Each regional center was responsible for participating women in early pregnancy in respective study areas. To maximize representativeness, baseline recruitment was performed in collaboration with local governments and local health care providers. The follow-up of the children from these pregnancies is still ongoing until they have reached 13 years of age.

**Figure 1.**
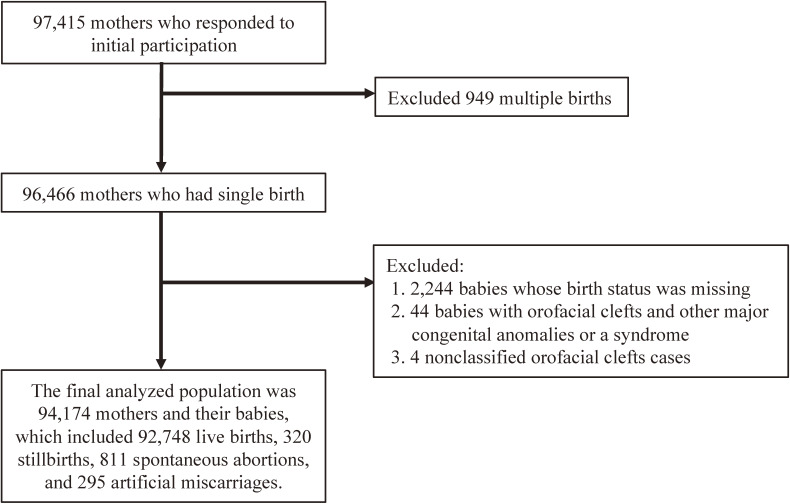
Flowchart of the study

The baseline recruitment targeted women in early pregnancy from January 2011 to March 2014. Figure 1 shows the flow chart of the current study. In the baseline survey, there were 103,062 pregnant cases by 97,415 mothers who responded to initial participation. Then, 96,466 mothers had single birth. We excluded 2,244 babies whose birth status was missing. To restrict to nonsyndromic cases, we excluded 44 babies with orofacial clefts and other major congenital anomalies or a syndrome; and then, 4 nonclassified orofacial clefts cases were also excluded. Thus, the final analyzed population was 94,174 pairs, which included 92,748 live births, 320 stillbirths, 811 spontaneous abortions, and 295 artificial miscarriages.

### Study design

This was a prospective cohort study.

### Dependent variable: nonsyndromic CL±P and CP

We defined nonsyndromic CL±P and CP as a dependent variable. Information on congenital anomalies was obtained from the medical record transcripts of babies at delivery and at 1 month of age. Details of processing, validation, and verification of the information on congenital anomalies were previously described elsewhere.^21^ The medical records included the information on cleft lip, cleft palate only, and cleft lip and palate. Based on the etiological distinction, we categorized these three types of orofacial clefts into CL±P and CP. Thus, cleft lip and cleft lip and palate were defined as CL±P, and cleft palate only were defined as CP.

Then, following the definition of congenital anomalies in JECS,^21^ we included cases that were diagnosed at either of the two periods. Cases with inconsistent diagnosis between delivery and 1 month of age were defined as nonclassified orofacial clefts.^21^ Since we excluded nonclassified orofacial clefts cases and babies with orofacial clefts and the other major congenital anomalies or any syndrome, all included cases were nonsyndromic.

### Independent variables

Based on the previous studies,^1^^,^^5^^–^^15^ we selected the following variables as potential risk factors for orofacial clefts: maternal alcohol consumption, psychological distress, maternal active smoking, maternal passive smoking, body mass index (BMI), and non-use of a folic acid supplement during pregnancy. The covariates were maternal age, sex of the baby, birth order, maternal educational attainment, and annual household income.

The self-administered questionnaires for the enrolled mothers were conducted at both 15 weeks of pregnancy (1st and 3rd quantile = 12 and 19) and 27 weeks of pregnancy (1st and 3rd quantile = 25 and 30). At 15 weeks of pregnancy, we obtained psychological distress, maternal active and passive smoking status, and status of intake of folic acid supplement during the first trimester. Psychological distress at 15 weeks of pregnancy was assessed using the Kessler Psychological Distress Scale (K6).^22^^–^^24^ We defined psychological distress by a cut-off point of 4/5 (none and having psychological distress).^22^^–^^24^ The information on maternal active and passive smoking at 15 weeks of pregnancy was obtained using the questionnaires. The categories of maternal active smoking status were defined as never smoker, former smoker who quit before pregnancy, former smoker who smoke during pregnancy and quit afterwards, and current smoker. The frequency of exposing passive smoking exposure was categorized as none, one to six times a week, and every day. The status of medication, drug, and supplement use was obtained through the interview by research coordinators at 15 weeks of pregnancy. If the participants used medication, drug, or supplement in the past year, they reported the name and time of administration. We obtained the status of intake of folic acid supplement during the first trimester: the categories were none or intake.

Maternal alcohol consumption during pregnancy was obtained using the Food Frequency Questionnaire at 27 weeks of pregnancy.^25^ We categorized maternal alcohol consumption during pregnancy as non-drinker during pregnancy, former drinker who quit after pregnancy, and current drinker. We also obtained maternal educational attainment (high school or lower, technical junior college or technical/vocational college, and university or higher) and annual household income (<4 million yen, 4 to 6 million yen, 6 to 8 million yen, and >8 million yen). Maternal age (<25, 25–29, 30–34, and ≥35 years old), BMI (underweight [<18.5 kg/m^2^], normal weight [18.5–25 kg/m^2^], and overweight [≥25 kg/m^2^]), and birth order (1st, 2nd, and ≥3rd) were transcribed from the medical records at 12 weeks of pregnancy (1st and 3rd quantile = 10 and 16). Sex of the baby was obtained from the medical records of babies at delivery (male or female).

### Statistical analysis

We conducted a logistic regression analysis to estimate odds ratios (ORs) for the birth prevalence of only nonsyndromic CL±P in babies with 95% confidence intervals (CIs). Because the number of CP cases was too small (*n* = 41), we did not estimate ORs for CP. The ORs can be interpreted as relative risks (RRs) because the birth prevalence of nonsyndromic CL±P was sufficiently small.^26^ In the fully adjusted models, psychological distress, maternal alcohol consumption, maternal active smoking, maternal passive smoking, BMI, intake of folic acid supplement, maternal age, sex of the baby, birth order, maternal educational attainment, and annual household income were included. We calculated crude and adjusted PAF and 95% CIs using the simple method with the Bonferroni inequality.^27^ If the upper or lower confidence interval of the PAF could not be calculated, it was indicated as not available (NA). In this study, PAF denotes the fraction of cases that would have occurred if exposure occurred, and the association is causal.^18^ We calculated PAFs of each category; then, we also dichotomized the categories of the risk factors to calculate a total PAF when a risk factor has more than two categories. We also estimated combined PAFs of modifiable risk factors for nonsyndromic CL±P, which indicates the sum of the PAFs of the modifiable risk factors.^28^ Besides, we also presented the birth prevalence of cleft lip (CL) and cleft lip and palate (CLP) among each variable in the eTable 1. Based on the assumption of missing at random, we conducted the k-nearest neighbor imputation method using the R package “DMwR”.^29^ The information on the missing values is shown in eTable 2. To confirm the validity of the results after imputation, we also conducted a logistic regression analysis with available-case analysis. Two-sided *P*-values of <0.05 were considered statistically significant. All analyses were conducted by R (version 3.5.2; R Foundation for Statistical Computing, Vienna, Austria) on macOS.

## RESULTS

The mean maternal age was 30.8 years, and the standard deviation was 5.1 years. The percentage of female babies was 48.6. There were 146 nonsyndromic CL±P (144 live births, 1 spontaneous abortion, and 1 artificial miscarriage) and 41 nonsyndromic CP cases (40 live births and 1 spontaneous abortion). The birth prevalence per 1,000 single births of nonsyndromic CL±P and CP were 1.55 and 0.44, respectively. Table 1 presents the characteristics and the birth prevalence of nonsyndromic CL±P and CP. Male babies had a high birth prevalence of nonsyndromic CL±P compared with female baby.

**Table 1.** Baseline characteristics and the birth prevalence of nonsyndromic orofacial clefts

		Total		Nonsyndromic CL±P		Nonsyndromic CP	
		(*n* = 94,174)		(*n* = 146)		(*n* = 41)	
		*n*	%	*n*	Birth prevalence (per 1,000 single births)	*n*	Birth prevalence (per 1,000 single births)
Maternal age, years	<25	10,114	11.5	17	1.68	3	0.30
	25–29	25,643	29.0	39	1.52	15	0.58
	30–34	30,738	34.8	51	1.66	12	0.39
	≥35	21,822	24.7	29	1.33	7	0.32
Sex of baby	Male	47,972	51.4	89	1.86	18	0.38
	Female	45,375	48.6	56	1.23	23	0.51
Birth order	1st	29,745	31.9	45	1.51	14	0.47
	2nd	30,820	33.0	55	1.78	15	0.49
	≥3rd	32,734	35.1	43	1.31	11	0.34
Maternal educational attainment	High school or lower	32,825	36.2	58	1.77	15	0.46
	Technical junior college or technical/vocational college	38,150	42.1	49	1.28	18	0.47
	University or higher	19,692	21.7	32	1.63	7	0.36
Annual household income	<4 million yen	33,942	40.1	50	1.47	12	0.35
	4–6 million yen	27,933	33.0	39	1.40	13	0.47
	6–8 million yen	13,496	16.0	22	1.63	10	0.74
	>8 million yen	9,226	10.9	15	1.63	4	0.43
Psychological distress	None	61,735	67.7	90	1.46	25	0.40
	Having	29,394	32.3	47	1.60	16	0.54
Maternal alcohol status	Nondrinker during pregnancy	45,191	50.0	76	1.68	29	0.64
	Former drinker who quit after pregnancy	42,744	47.3	63	1.47	10	0.23
	Current drinker	2,509	2.8	3	1.20	1	0.40
Maternal active smoking	Never smoker	53,309	58.2	74	1.39	26	0.49
	Former smoker who quit before pregnancy	21,384	23.4	38	1.78	10	0.47
	Former smoker who smoke during pregnancy and quit afterwards	12,425	13.6	24	1.93	4	0.32
	Current smoker	4,419	4.8	6	1.36	1	0.23
Maternal passive smoking	None	45,212	49.3	60	1.33	25	0.55
	One to six times a week	29,525	32.2	47	1.59	12	0.41
	Every day	17,041	18.6	35	2.05	4	0.23
Body Mass Index, kg/m^2^	Underweight (<18.5)	10,321	11.2	17	1.65	4	0.39
	Normal weight (18.5–25)	68,761	74.9	104	1.51	29	0.42
	Overweight (≥25)	12,751	13.9	22	1.73	7	0.55
Folic acid supplementation	Intake	25,529	27.1	34	1.33	15	0.59
	None	68,645	72.9	112	1.63	26	0.38

CL±P, cleft lip with or without cleft palate; CP, cleft palate only.

Psychological distress, maternal active and passive smoking, folic acid supplementation were obtained at 15 weeks of pregnancy.

Body mass index, maternal age, and birth order were obtained at 12 weeks of pregnancy.

Maternal alcohol status, maternal educational attainment, and annual household income were obtained at 27 weeks of pregnancy.

Table 2 shows the associations of the risk factors with CL±P, and the PAFs after imputation. In the crude models, only maternal former and current alcohol drinkers during the pregnancy and current smokers were not associated with CL±P risk. In the fully adjusted models, the combined adjusted PAF of the modifiable risk factors for CL±P, excluding maternal alcohol consumption, was 34.3%. Compared with nondrinkers during pregnancy, former drinkers who quit after pregnancy and current drinkers were not associated with CL±P risk (OR of former drinker who quit after pregnancy, 0.87; 95% CI, 0.63–1.22 and OR of current drinker, 0.71; 95% CI, 0.22–2.27). The total adjusted PAF of psychological distress for CL±P was 1.4% (95% CI, −10.7 to 15.1%). The total adjusted PAF of maternal smoking status for CL±P was 9.9% (95% CI, −7.0 to 26.9%): the PAF of former smoker who quit before pregnancy was 5.5% (95% CI, −4.3 to 17.8%), the PAF of former smoker who smoke during pregnancy and quit afterwards was 3.7% (95% CI, −3.3 to 14.9%), and the PAF of current smoker was −0.9% (95% CI, NA to 7.6%). The total adjusted PAF of maternal passive smoking was 10.8% (95% CI, −9.9 to 30.3%): the PAF of one to six times a week was 4.4% (95% CI, −8.1 to 18.8%) and the PAF of every day was 7.9% (95% CI, −2.1 to 20.5%). The total adjusted PAF of BMI was 2.4% (95% CI, −7.5 to 14.0%): the PAF of underweight was 0.9% (95% CI, −3.2 to 9.6%) and the PAF of overweight was 1.5% (95% CI, −4.4 to 10.9%). The total adjusted PAF of non-use of a folic acid supplement during the first trimester was 15.1% (95% CI, −17.8 to 41.0%), which was the highest in this study. The results from the models after imputation were similar to those in the available-case analysis (eTable 3).

**Table 2.** Associations of the modifiable risk factors with nonsyndromic CL±P and the population attributable fractions after imputation

		Prevalence among cases (%)	Nonsyndromic CL±P											

			Crude Model						Fully adjusted model^a^					
			(*n* = 94,174)						(*n* = 94,174)					
	
			OR	95% CI	PAF (%)	95% CI	Total crude PAF of each risk factor (%)	95% CI	OR	95% CI	PAF (%)	95% CI	Total adjusted PAF of each risk factor (%)	95% CI
Psychological distress (reference: none)	Having	33.6	1.08	0.76, 1.52	2.4	−9.5, 15.9	2.4	−9.5, 15.9	1.05	0.74, 1.48	1.5	−10.7, 15.2	1.4	−10.7, 15.1
Maternal alcohol status (reference: nondrinker during pregnancy)	Former drinker who quit after pregnancy	45.9	0.93	0.67, 1.29	−3.6	−21.1, 14.5	−4.3	−22.7, 14.3	0.87	0.63, 1.22	−6.6	−25.1, 12.3	−7.3	−26.7, 12.2
	Current drinker	2.1	0.74	0.23, 2.34	−0.7	NA, 7.6			0.71	0.22, 2.27	−0.8	NA, 7.4		
Maternal smoking status (reference: never smoker)	Former smoker who quit before pregnancy	26.0	1.25	0.84, 1.84	5.1	−4.3, 17.2			1.27	0.85, 1.90	5.5	−4.3, 17.8		
	Former smoker who smoke during pregnancy and quit afterwards	17.1	1.43	0.91, 2.24	5.1	−1.4, 15.5	10.1	−5.4, 26.1	1.28	0.77, 2.12	3.7	−3.3, 14.9	9.9	−7.0, 26.9
	Current smoker	4.1	0.96	0.42, 2.21	−0.2	NA, 8.1			0.82	0.34, 1.99	−0.9	NA, 7.6		
Maternal passive smoking (reference: none)	At least one day a week	32.9	1.17	0.81, 1.71	4.9	−7.2, 19.0	13.1	−5.7, 31.2	1.15	0.78, 1.70	4.4	−8.1, 18.8	10.8	−9.9, 30.3
	Every day	24.0	1.52	1.01, 2.30	8.2	−0.8, 19.9			1.49	0.93, 2.39	7.9	−2.1, 20.5		
Body mass index (kg/m^2^) (reference: normal weight [18.5–25])	Underweight (<18.5)	11.6	1.08	0.65, 1.80	0.8	−3.2, 9.6	2.7	−7.0, 14.3	1.08	0.65, 1.81	0.9	−3.2, 9.6	2.4	−7.5, 14.0
	Overweight (≥25)	15.1	1.14	0.72, 1.81	1.9	−3.9, 11.2			1.11	0.70, 1.76	1.5	−4.4, 10.9		
Folic acid supplementation (reference: intake)	None	76.7	1.23	0.83, 1.80	14.1	−18.6, 40.1	14.1	−18.6, 40.1	1.25	0.84, 1.84	15.2	−17.6, 41.1	15.1	−17.8, 41.0
Combined PAF^b^							36.3						34.3	

CI, confidence interval; CL±P, cleft lip with or without cleft palate; NA, not available; OR, odds ratio; PAF, population-attributable fraction.

^a^In the fully adjusted model, maternal age, sex of the baby, birth order, maternal educational attainment, annual household income, psychological distress, maternal alcohol status, maternal active and passive smoking, body mass index, and folic acid supplementation were adjusted.

^b^Combined PAF included psychological distress, maternal active and passive smoking, body mass index, and non-use of a folic acid supplement.

Psychological distress, maternal active and passive smoking, folic acid supplementation were obtained at 15 weeks of pregnancy.

Body mass index, maternal age, and birth order were obtained at 12 weeks of pregnancy.

Maternal alcohol status, maternal educational attainment, and annual household income were obtained at 27 weeks of pregnancy.

The imputation was conducted using the k nearest neighbors imputation method.

If the upper or lower confidence interval of the PAF could not be calculated, it was indicated as “NA.”

## DISCUSSIONS

This prospective cohort study presented the PAFs of the modifiable risk factors for nonsyndromic CL±P. Among CL±P cases, non-use of a folic acid supplement during the first trimester had the highest PAF (15.1%), followed by maternal active and passive smoking (9.9% and 10.8%, respectively). However, we could not calculate PAFs for CP due to the small sample size.

The limitations of the current study should be noted. First, we failed to obtain a statistically significant association, despite conducting a nationwide birth cohort study with a large sample size. Among CL±P, we obtained wider CIs, indicating a high probability of false-negative associations. Second, we did not consider the effects of the genetic risk factors for orofacial clefts. Previous studies reported the complex interaction between multiple lifestyle, environmental, and genetic factors.^1^^,^^5^ Especially, several studies reported the gene–environment interactions of CL±P.^5^^,^^30^^,^^31^ For example, the effects of maternal smoking on CL±P were modified by the genes in the detoxification pathways, and it might have an important role in the etiology of CL±P.^5^^,^^30^ Alcohol metabolism genes have also been recognized as the interacting factor of maternal alcohol consumption for CL±P.^5^^,^^31^ Therefore, in this study, although the participants had risk factors, some of them might have a low risk of CL±P due to the interaction of genes.^5^^,^^30^^,^^31^ Further study, including genetic information, is needed to estimate PAFs more accurately. Besides, the orofacial clefts history of parents was not included in this study. Family history of orofacial clefts strongly predicts the recurrence in newborns.^19^^,^^32^ Parents with orofacial clefts can avoid exposure to known modifiable factors to prevent reoccurrence in their babies. Therefore, this limitation might weaken the associations of the known risk factors of orofacial clefts. However, most results of CL±P were consistent with those from the previous studies. Besides, because several previous studies had case-control design, these could include the effects of recall bias. The present cohort study suggests a new insight of prospective associations and supports the existing evidence for CL±P.

Although the previous study assessed the association of maternal binge alcohol consumption during the pregnancy, there was no strong association: the pooled ORs of binge alcohol consumption were 1.04 (95% CI, 0.87–1.24) for CL±P.^13^ In this study, maternal alcohol consumption during the pregnancy was inversely associated with CL±P risk. As the studies pointed out the existence of unknown biases,^13^ this result can also be biased. In Japan, the effects of alcohol consumption during the pregnancy are the well-known risk factors for the babies’ health because pregnant women have been educated on lifestyle habits during pregnancy.^33^ Most parents with orofacial clefts might tend to avoid drinking alcohol to reduce the recurrent risk. Therefore, there could be a high birth prevalence of orofacial clefts among healthy behavior groups. It is difficult to interpret the associations of maternal alcohol consumption with orofacial clefts based only on our results.

We observed that maternal active smoking was associated with a high risk of CL±P. The reviews indicated low- or modest-quality evidence of dose-response effects of maternal active smoking in CL±P.^6^^,^^7^ The two studies reported that the ORs of maternal active smoking during pregnancy were 1.37 (95% CI, 1.26–1.49) and 1.34 (95% CI, 1.25–1.44) for CL±P.^6^^,^^7^ The current study showed a relatively similar OR of maternal active smoking for CL±P (OR of former smoker who quit before pregnancy, 1.27; 95% CI, 0.85–1.90 and OR of former smoker who smoke during pregnancy and quit afterwards, 1.28; 95% CI, 0.77–2.12), excluding current smoking (OR 0.82; 95% CI, 0.34–1.99). We expected that current smokers had the highest risk of CL±P; however, the current results showed no increased association between current smoking and CL±P. This difference might be a self-reported bias that active smoking mothers may underreport the real status due to societal norms.^34^ There is a possibility of underestimation of current active smoking effects. However, compared with the previous study in the United States,^19^ our study reported higher PAFs of maternal active smoking (9.9% vs 3.99%). Maternal active smoking can be a large burden on CL±P, especially in Japan.

To the best of our knowledge, this is the first study that reported the PAFs of maternal passive smoking in orofacial clefts. A previous study reported that maternal passive smoking increased the risk of CL±P (OR 2.05; 95% CI, 1.27–3.30).^8^ The previous study in the United States assessed the population impact of only maternal active smoking on orofacial clefts, but not passive smoking.^19^ In this study, maternal passive smoking had the second highest PAF, and there seems to be a dose-response association in CL±P (OR of exposed at least one day a week, 1.15; 95% CI, 0.78–1.70 and OR of exposed every day, 1.49; 95% CI, 0.93–2.39). Although the Japanese Ministry of Health, Labour and Welfare decided to conduct a smoking ban in public indoor spaces,^34^ many individuals continue to be exposed to secondhand smoke.^35^ Besides, the previous study reported that several individuals in Japan could not avoid exposure to secondhand smoke at the workplace and in school, even if they have knowledge of tobacco’s adverse health effects.^35^ Indeed, the prevalence of maternal passive smoking was relatively high. Maternal passive smoking can be associated with a relatively large proportion of CL±P cases.

We found that the PAF of non-use of a folic acid supplement during the first trimester was highest in this study. The Cochrane review reported low-quality evidence of the preventive effects of folic acid supplementation on cleft lip (RR 0.79; 95% CI, 0.14–4.36) and cleft palate (RR 0.73; 95% CI, 0.05–10.89).^36^ The Japanese Ministry of Health, Labour and Welfare recommends folic acid supplementation during pregnancy.^37^ However, in Japan, folic acid supplementation is not popular, but many pregnant women know the positive effects of folic acid supplementation on neural tube defects.^37^ In this study, the percentage of pregnant women receiving folic acid supplementation in Japan was also relatively low (prevalence was 27.1%). The PAF of non-use of a folic acid supplement for CL±P in this study was larger than that in the United States (15.1% vs 3.34%).^19^ This result can support recommendations for folic acid supplementation in Japan. However, there was no information on scheduled pregnancy, which affects status of the folic acid supplement intake before pregnancy and can produce the bias. Besides, as we could not obtain the information on the dose of folic acid in supplements, the amount of folic acid received during supplementation was unclear. Intake of folic acid over 1000 µg/day is not recommended.^37^ The effects of higher dose of folic acid in supplements should be noted.

In this study, overweight and underweight were weakly associated with CL±P (OR of overweight, 1.11; 95% CI, 0.70–1.76 and OR of underweight, 1.08; 95% CI, 0.65–1.81). Previous studies reported that obesity was weakly associated with risk for CL±P (OR 1.16; 95% CI, 1.00–1.34), while the risk of orofacial clefts in overweight was extremely small (OR for CL±P, 1.06; 95% CI, 0.93–1.21).^12^ Japan has the lowest prevalence of obesity among the Organization for Economic Cooperation and Development countries (4.2%).^38^ Indeed, as the prevalence of overweight was also relatively low, the PAFs of overweight were small. In contrast, few studies focused on the associations of underweight with orofacial clefts. These studies reported a weak association between maternal underweight and orofacial clefts in babies.^39^^,^^40^ In this study, the PAF of underweight for CL±P was also small.

Although previous studies suggested the association of maternal stressful life events with the high risk of orofacial clefts through stress-induced corticosteroids,^9^ there is no study that directly measured maternal psychological distress. Our study measured nonspecific psychological distress using the K6 and might support the associations of CL±P.

### Conclusions

This prospective cohort study shows the association of the modifiable risk factors with CL±P and the population impact in Japan. We could not show associations of CP, although the total sample size is large. Non-use of a folic acid supplement had the highest PAF for CL±P (15.1%; 95% CI, −17.8 to 41.0%), followed by maternal active and passive smoking (9.9%; 95% CI, −7.0 to 26.9% and 10.8%; 95% CI, −9.9 to 30.3%, respectively). We failed to obtain significant associations between variables, but these results support the previous evidence. This potential population impact of the modifiable risk factors might be useful for policymakers and health workers to plan the primary prevention of orofacial clefts. For example, smoking ban in public places could reduce the prevalence of maternal active and passive smoking,^41^^,^^42^ and such policies might reduce the incidence of orofacial clefts. Besides, previous studies reported that the compulsory folic acid fortification in cereal grain products could reduce the incidence of orofacial clefts in the United States.^43^^,^^44^ The interventions on policies of folic acid fortification and smoking ban might help prevent CL±P. Besides, as health behavior change interventions such as health coaching has succeeded in reducing weight gain among pregnant women,^45^^,^^46^ such interventions might also be useful to reduce CL±P.

## APPENDIX A. SUPPLEMENTARY DATA

eTable 1. Baseline characteristics and birth prevalence of nonsyndromic CLP and CL

eTable 2. Information on the missing values

eTable 3. Associations of the modifiable risk factors with nonsyndromic CL±P and the population attributable fractions using available-case analysis
