# Supplementary material for: Population Attributable Fractions of Modifiable Risk Factors for Nonsyndromic Orofacial Clefts: A Prospective Cohort Study From the Japan Environment and Children’s Study
Source: J Epidemiol. 2021 Apr 5;31(4):272–9. doi: 10.2188/jea.JE20190347 (PMC7940975; doi:10.2188/jea.JE20190347)
Supplement: Supplementary file 1 [file je-31-272-s001.pdf]

eTable 1. Baseline characteristics and birth prevalence of nonsyndromic CLP and CL

		Total (n= 94,174)		Nonsyndromic CLP (n= 80)		Nonsyndromic CL (n= 59)	
		n	%	n	Birth prevalence (per 1,000 single live births)	n	Birth prevalence (per 1,000 single live births)
Maternal age, years	<25	10,114	11.5	10	0.99	5	0.49
	25–29	25,643	29.0	26	1.01	12	0.47
	30–34	30,738	34.8	27	0.88	23	0.75
	≥35	21,822	24.7	12	0.55	14	0.64
Sex of baby	Male	47,972	51.4	53	1.10	33	0.69
	Female	45,375	48.6	27	0.60	25	0.55
Birth order	1st	29,745	31.9	29	0.97	14	0.47
	2nd	30,820	33.0	28	0.91	24	0.78
	≥3rd	32,734	35.1	21	0.64	20	0.61
Maternal educational attainment	High school or lower	32,825	36.2	37	1.13	18	0.55
	Technical junior college or technical/vocational college	38,150	42.1	20	0.52	27	0.71
	University or higher	19,692	21.7	20	1.02	10	0.51
Annual household income	<4 million yen	33,942	40.1	24	0.71	24	0.71
	4–6 million yen	27,933	33.0	24	0.86	13	0.47
	6–8 million yen	13,496	16.0	15	1.11	7	0.52
	>8 million yen	9,226	10.9	8	0.87	5	0.54
Psychological distress	None	61,735	67.7	51	0.83	37	0.60
	Having	29,394	32.3	25	0.85	18	0.61

Maternal alcohol status	Nondrinker during pregnancy	45,191	50.0	47	1.04	26	0.58
	Former drinker who quit after pregnancy	42,744	47.3	31	0.73	29	0.68
	Current drinker	2,509	2.8	1	0.40	1	0.40
Maternal active smoking	Never smoker	53,309	58.2	42	0.79	29	0.54
	Former smoker who quit before pregnancy	21,384	23.4	21	0.98	17	0.79
	Former smoker who smoke during pregnancy and quit afterwards	12,425	13.6	15	1.21	7	0.56
	Current smoker	4,419	4.8	1	0.23	4	0.91
Maternal passive smoking	None	45,212	49.3	33	0.73	26	0.58
	One to six times a week	29,525	32.2	28	0.95	17	0.58
	Every day	17,041	18.6	18	1.06	14	0.82
Body Mass Index, kg/m^2	Underweight (<18.5)	10,321	11.2	11	1.07	6	0.58
	Normal weight (18.5–25)	68,761	74.9	55	0.80	45	0.65
	Overweight (≥25)	12,751	13.9	13	1.02	6	0.47
Folic acid supplementation	Intake	25,529	27.1	26	1.02	8	0.31
	None	68,645	72.9	54	0.79	51	0.74

CL, cleft lip; CLP, cleft lip and palate.

Psychological distress, maternal active and passive smoking, folic acid supplementation were obtained at 15 weeks of pregnancy.

Body mass index, maternal age, and birth order were obtained at 12 weeks of pregnancy.

Maternal alcohol status, maternal educational attainment, and annual household income were obtained at 27 weeks of pregnancy.

eTable 2. Information on the missing values

Variables	Number of missing n	%	Types of variable in the imputation (Categorical or continuous variables)
Maternal age	5,857	6.2	Categorical
Sex of the baby	827	0.9	Categorical
Birth order	875	0.9	Categorical
Maternal educational attainment	3,507	3.7	Categorical
Annual household income	9,577	10.2	Categorical
Psychological distress	3,045	3.2	Categorical
Maternal alcohol status	3,730	4.0	Categorical
Maternal active smoking	2,637	2.8	Categorical
Maternal passive smoking	2,396	2.5	Categorical
Body mass index	2,341	2.5	Categorical
Folic acid supplementation	0	0.0	Categorical
Orofacial clefts	0	0.0	Categorical

The weighted average methods were used, and the number of neighbors was defined as 5.

eTable 3. Associations of the modifiable risk factors with nonsyndromic CL±P and the population attributable fractions using available-case analysis

			Nonsyndromic CL±P											
			Crude Model						Fully adjusted model[a] (n = 74,860)					
		Prevalence among cases (%)	OR	95% CI	PAF (%)	95% CI	Total crude PAF of each risk factor	95% CI	OR	95% CI	PAF (%)	95% CI	Total adjusted PAF of each risk factor	95% CI
Psychological distress (reference: none)	Having	34.3	1.10	0.77, 1.56	3.0	-9.3, 16.9	3.0	-9.3, 16.9	1.11	0.75, 1.66	3.5	-10.5, 18.6	3.6	-10.4, 18.6
Maternal alcohol status (reference: nondrinker during pregnancy)	Former drinker who quit after pregnancy	44.4	0.88	0.63, 1.22	-6.3	-23.7, 12.0	-7.1	-25.5, 11.7	0.80	0.54, 1.18	-10.9	-33.2, 10.8	-11.4	-34.6, 10.9
	Current drinker	2.1	0.71	0.22, 2.25	-0.9	NA, 7.4			0.82	0.25, 2.64	-0.5	0.0, 8.0		
Maternal smoking status (reference: never smoker)	Former smoker who quit before pregnancy	26.8	1.28	0.87, 1.89	5.9	-3.9, 18.2	10.5	-5.3, 26.8	1.27	0.80, 2.01	5.6	-5.9, 19.4	10.2	-9.8, 29.1
	Former smoker who smoke during pregnancy and quit afterwards	16.9	1.39	0.88, 2.21	4.8	-1.7, 15.2			1.23	0.67, 2.27	3.2	-5.0, 15.8		
	Current smoker	4.2	0.98	0.43, 2.25	-0.1	NA, 8.3			1.22	0.49, 3.05	0.8	0.0, 9.8		
Maternal passive smoking (reference: none)	At least one day a week	33.1	1.20	0.82, 1.76	5.5	-7.0, 20.1	14.2	-5.0, 32.6	1.14	0.73, 1.77	4.0	-10.8, 20.4	9.4	-15.4, 31.4
	Every day	24.6	1.55	1.02, 2.35	8.7	-0.6, 21.0			1.36	0.78, 2.36	6.5	-6.1, 21.4		
Body mass index (kg/m^2) (reference: normal weight [18.5–	Underweight (<18.5)	11.9	1.09	0.65, 1.82	1.0	-3.2, 9.9	2.9	-7.1, 14.6	1.36	0.79, 2.33	3.1	-1.8, 12.2	4.8	-6.3, 17.2

25])														
	Overweight (≥25)	15.4	1.14	0.72, 1.81	1.9	-4.1, 11.4			1.09	0.63, 1.88	1.3	-5.9, 12.0		
Folic acid supplementation (reference: intake)	None	76.7	1.23	0.83, 1.80	14.1	-18.6, 40.1	14.1	-18.6, 40.1	1.32	0.85, 2.07	18.7	-18.1, 46.1	18.7	-18.1, 46.1
Combined PAF[b]							37.9						39.3	

CI, confidence interval; CL±P, cleft lip with or without cleft palate; NA, not available; OR, odds ratio; PAF, population-attributable fraction.

[a]In the fully adjusted model, maternal age, sex of the baby, birth order, maternal educational attainment, annual household income, psychological distress, maternal alcohol status, maternal active and passive smoking, body mass index, and folic acid supplementation were adjusted.

[b]Combined PAF included psychological distress, maternal active and passive smoking, body mass index, and non-use of a folic acid supplement.

Psychological distress, maternal active and passive smoking, folic acid supplementation were obtained at 15 weeks of pregnancy.

Body mass index, maternal age, and birth order were obtained at 12 weeks of pregnancy.

Maternal alcohol status, maternal educational attainment, and annual household income were obtained at 27 weeks of pregnancy.

If the upper or lower confidence interval of the PAF could not be calculated, it was indicated as “NA.”
